# Predictive nomogram models for unfavorable prognosis after aneurysmal subarachnoid hemorrhage: Analysis from a prospective, observational cohort in China

**DOI:** 10.1111/cns.14288

**Published:** 2023-06-08

**Authors:** Sijia Li, Jia Zhang, Ning Li, Dandan Wang, Xingquan Zhao

**Affiliations:** ^1^ Department of Neurology, Beijing Tiantan Hospital Capital Medical University Beijing China; ^2^ China National Clinical Research Center for Neurological Diseases Beijing China; ^3^ Research Unit of Artificial Intelligence in Cerebrovascular Disease Chinese Academy of Medical Sciences Beijing China; ^4^ Center of Stroke, Beijing Institute of Brain Disorders Capital Medical University Beijing China

**Keywords:** aneurysmal subarachnoid hemorrhage, nomogram, prediction, prognosis

## Abstract

**Aim:**

The aim of the study was to identify predictors for 3‐month poor functional outcome or death after aSAH and develop precise and easy‐to‐use nomogram models.

**Methods:**

The study was performed at the department of neurology emergency in Beijing Tiantan Hospital. A total of 310 aSAH patients were enrolled between October 2020 and September 2021 as a derivation cohort, while a total of 208 patients were admitted from October 2021 to March 2022 as an external validation cohort. Clinical outcomes included poor functional outcome defined as modified Rankin Scale score (mRS) of 4–6 or all‐cause death at 3 months. Least absolute shrinkage and selection operator (LASSO) analysis, as well as multivariable regression analysis, were applied to select independent variables associated with poor functional outcome or death and then to construct two nomogram models. Model performance were evaluated through discrimination, calibration, and clinical usefulness in both derivation cohort and external validation cohort.

**Results:**

The nomogram model to predict poor functional outcome included seven predictors: age, heart rate, Hunt‐Hess grade on admission, lymphocyte, C‐reactive protein (CRP), platelet, and direct bilirubin levels. It demonstrated high discrimination ability (AUC, 0.845; 95% CI: 0.787–0.903), satisfactory calibration curve, and good clinical usefulness. Similarly, the nomogram model combining age, neutrophil, lymphocyte, CRP, aspartate aminotransferase (AST) levels, and treatment methods to predict all‐cause death also revealed excellent discrimination ability (AUC, 0.944; 95% CI: 0.910–0.979), satisfactory calibration curve, and clinical effectiveness. Internal validation showed the bias‐corrected C‐index for poor functional outcome and death was 0.827 and 0.927, respectively. When applied to the external validation dataset, both two nomogram models exhibited high discrimination capacity [poor functional outcome: AUC = 0.795 (0.716–0.873); death: AUC = 0.811 (0.707–0.915)], good calibration ability, and clinical usefulness.

**Conclusions:**

Nomogram models constructed for predicting 3‐month poor functional outcome or death after aSAH are precise and easily applicable, which can help physicians to identify patients at risk, guide decision‐making, and provide new directions for future studies to explore the novel treatment targets.

## Graphical abstract

We constructed precise and easily applicable nomogram models to predict 3‐month poor functional outcome or death after aSAH. These prediction models can help physicians to identify patients at risk, guide decision‐making, and provide new directions for future studies to explore the novel treatment targets.

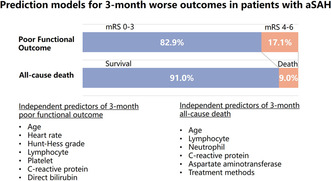

## 1 INTRODUCTION

Aneurysmal spontaneous subarachnoid hemorrhage (aSAH) is a severe type of stroke, usually affecting young and healthy adults with a sudden dramatic onset.^1^, ^2^ The mortality rate of aSAH is up to 35% and approximately one‐third of survivors suffered from severe disability and functional dependence throughout their resting of life.^3^ Due to the high mortality and low functional independent rate of aSAH, it is crucial to predict the prognosis promptly and accurately instead of relying on clinical intuition.^4^ Clinical prediction models, with a combination of various characteristics of patients and disease, can serve as useful tools to evaluate individuals' outcomes, guide decision‐making about targeted therapy, and facilitate effective communications with patients and their family members.

Several clinical prediction tools have been applied to evaluate the outcomes in patients with aSAH. The Hunt‐Hess grade, WFNS grade, and modified Fisher grade have been widely used to predict outcomes and help make clinical decisions.^5^, ^6^, ^7^ However, these three grading systems do not incorporate other clinical parameters like age and comorbidities.^8^ Also, evaluating prognosis based on these systems is somewhat subjective because of the ambiguous description between adjacent scales.^9^ Therefore, researchers have devoted themselves to exploring models that combine various factors to better predict the prognosis. Recently, the Subarachnoid Hemorrhage International Trialists (SAHIT) group developed three models (a core, neuroimaging, and full model) based on pooled randomized controlled trial data to predict death or functional outcome after 3 months in patients with aSAH.^4^ The core SAHIT models incorporate age, premorbid hypertension, and WFNS grade, which performed well and demonstrated good accuracy in an external validation cohort.^4^ Nonetheless, applying SAHIT models is difficult to some extent due to some poorly defined predictors like hypertension and their inconvenient use with an online calculator.^3^, ^9^ Furthermore, predictors in these models just partially explained the variability in outcomes, indicating that other factors may have an impact on outcome prediction. Hence, it is crucial to incorporate other new, objective and readily available predictors to establish easy‐to‐use clinical prediction models in order to guide targeted treatment and provide inspirations for future studies. Among the computational models to predict outcomes, the nomogram is a valuable and user‐friendly prediction tool, since it can present a model graphically and have the advantage to accurately calculate the probability of a clinical event compared with the conventional odd ratios (ORs).^10^

Therefore, based on this modern‐day, observational cohort, we aimed to identify new predictors for 3‐month poor functional outcome or all‐cause death and develop two novel nomogram models.

## 2 METHODS

### 2.1 Patients

The study was a prospective, observational cohort study of aSAH patients admitted to the department of neurology emergency in Beijing Tiantan Hospital. The derivation cohort comprised eligible aSAH patients admitted between October 2020 and September 2021, while the external validation cohort was composed of aSAH patients enrolled from October 2021 to March 2022. The study was performed in compliance with the ethical guidelines from the Helsinki Declaration and was approved by the Institutional Review Board of Beijing Tiantan Hospital. Written informed consent was obtained from all the participants or their legal proxies. The inclusion criteria were (1) age of 18 years or older, (2) spontaneous SAH diagnosed via head CT scan, (3) presence of intracranial aneurysm confirmed by CT angiopathy (CTA), or digital subtraction angiopathy (DSA). The exclusion criteria encompassed: (1) SAH caused by other reasons, such as trauma, cerebral arteriovenous malformations, moyamoya disease, and intracranial tumors, (2) previous history of cerebral infarction, intracranial hemorrhage, vascular anomalies, and malformations, (3) acute kidney injury or chronic kidney disease, (4) concurrent systemic comorbidities including malignancy and liver cirrhosis, (5) premorbid modified Rankin Scale (mRS) >1.

### 2.2 Data collection

Clinical information including demographic data (sex and age), previous history (hypertension, diabetes mellitus, coronary heart disease, current smoking, and drinking), neurological status, radiological characteristics, and laboratory tests on admission were all collected. Neurological status on admission were assessed by Hunt‐Hess grade and World Federation of Neurosurgical Societies (WFNS) grade. Radiological characteristics included modified Fisher grade, the number (“single” or “multiple”) of aneurysms, the location (“anterior cerebral artery,” “middle cerebral artery,” “internal carotid artery,” and “posterior circulation”) of the aneurysms,^4^ and the morphology of aneurysms (“single‐sac aneurysms with smooth margin,” “single‐sac aneurysms with irregular margin,” “aneurysms with a daughter sac,” and “multilobulated aneurysms”).^11^

Blood samples were drawn from an antecubital vein within 30 min upon arrival and prior to any treatment performed. Laboratory tests, including white blood cell (WBC), lymphocyte, monocyte, neutrophil, red blood cell (RBC), hemoglobin (Hb), platelet (PLT), C‐reaction protein (CRP), fibrin degeneration products (FDP), D‐dimer, fibrinogen (Fbg), prothrombin time (PT), activated partial thromboplastin time (APTT), thrombin time (TT), potassium, sodium, chlorine, glucose, blood urea nitrogen (BUN), creatinine (Cr), estimated glomerular filtration rate (eGFR), alanine aminotransferase (ALT), aspartate aminotransferase (AST), albumin (ALB), total bilirubin (TBIL), direct bilirubin (DBIL), indirect bilirubin (IBIl), creatine kinase isoenzyme MB (CK‐MB), and cardiac troponin I (cTNI), were collected.

Treatment modalities, including surgical clipping, endovascular coiling, or conservative treatment, were determined by the professional and experienced endovascular specialists and neurosurgeons according to the condition of each patient under the guidance of the current guidelines.^12^ In addition, willing of the patients' family were also taken into fully consideration.

All treatment methods were not at the discretion of our researchers, and patients in our study were not predefined to a certain group of an intervention or exposure. Also, there was no experimental therapy performed on enrolled patients and no biospecimens were additionally extracted or retained for further investigation. Therefore, our study was a purely observational study with no‐trial designs. According to the clinical registration requirements recommended by the International Committee Medical Journal Editors (ICMJE), a purely observational study does not require to register.^13^, ^14^ Hence, the clinical registration information for the present study was waived.

### 2.3 Outcomes

All the patients were followed up through telephone interviews at 3 months after symptom onset by trained interviewers who were blinded to baseline characteristics and prognostic factors. Clinical outcomes included unfavorable functional outcome defined as mRS score of 4–6 or all‐cause death at 3 months.^4^

### 2.4 Sample size

Currently, the events per variable (EPV) criterion, especially an EPV of 10, is widely used as the lowest limit for logistics regression models to predict a binary outcome.^15^ A total of seven variables were finally included in the multivariable logistics regression analysis to predict poor functional outcome. Therefore, the effective sample size of the derivation cohort should be at least 70 patients. Additionally, considering the occurrence of poor functional outcome after aSAH is around 30% worldwide,^3^ there should be at least 233 patients in the derivation cohort.

### 2.5 Statistics analysis

Statistics analysis was performed using IBM SPSS Statistics for Windows, version 22.0 (IBM Corp, Armonk, NY, USA) and R software (https://www.r‐project.org/, version 4.1.2). Shapiro–Wilk test was conducted to check whether the included variables were normally distributed. Continuous variables are expressed as means ± SD or medians with interquartile range according to the distribution of the data. Categorical variables are described as numbers (percentages).

Concerning the limited sample size of our study, LASSO regression, which has the advantage to analyze high‐dimensional data,^16^ was performed to select the potential prognostic factors for unfavorable functional outcome and death, respectively. Features with nonzero coefficients in the LASSO regression were then entered into multivariable logistics regression models. Backward Wald selection method was applied to obtain the independent prognostic factors. Finally, nomogram models in the two dimensions of poor functional outcome and all‐cause death were built based on the independent factors in logistics regression models respectively.

Model performance was assessed in terms of discrimination capacity, calibration ability, and clinical effectiveness. The area under the receiver operating characteristics (ROC) curve (AUC) was applied to evaluate the discrimination capacity of the nomogram models. DeLong's test was performed to compare the discriminative capacity between the nomogram models and the core SAHIT model. Calibration curves were plotted to show the agreement between the nomogram models and the expected observation. A plot near the 45‐degree line would implicate the perfect calibration ability of the model. Furthermore, decision curve analysis was performed to assess the clinical effectiveness of the models by calculating the net benefits at different threshold possibilities.^17^ Moreover, bootstrapping method (1000 bootstrap resamples) was applied to validate the models internally and a corrected C‐index was also calculated. In addition, the model performance in terms of discrimination, calibration and clinical effectiveness was also assessed using the external validation cohort.

We used the “Matrix” and “glmnet” package to generate the LASSO regression results. The “SparseM,” “ggplot2,” “Formula,” “survival,” “lattice,” “Hmisc,” and “rms” package were applied to build nomogram models, generate the C‐index and calibration curves. In addition, the “pROC” and “rmda” package were performed to plot ROC curves and decision curves, respectively. All tests of significance were two‐tailed, and *p*‐values <0.05 were considered statistically significant.

## 3 RESULTS

### 3.1 Characteristics of patients in derivation cohort

A total of 310 patients diagnosed with aSAH were included in derivation cohort. Among them, 64.5% (200) were females and the mean age was 56.1 ± 12.4 years old. Unfavorable functional outcome after 3 months was observed in 17.1% of the patients, while 3‐month all‐cause death was found in 9.0% of the patients. The baseline characteristics of patients in derivation cohort grouped by 3‐month poor functional outcome or all‐cause death are shown in Table 1 and Table S1, respectively.

**TABLE 1:** Baseline characteristics of the patients in derivation cohort grouped by 3‐month functional outcome.

	Total (*n* = 310)	Good functional outcome (*n* = 257)	Poor functional outcome (*n* = 53)	*p*
Age (years)	56.1 ± 12.4	54.5 ± 11.9	63.8 ± 11.9	<0.01
Female sex	200 (64.5)	166 (64.6)	34 (64.2)	0.95
History				
Hypertension	191 (61.6)	155 (60.3)	36 (67.9)	0.30
Diabetes mellitus	31 (10.0)	24 (9.3)	7 (13.2)	0.39
Coronary heart disease	25 (8.1)	20 (7.8)	5 (9.4)	0.78
Current smoking	53 (17.1)	48 (18.7)	5 (9.4)	0.10
Alcohol	42 (13.5)	37 (14.4)	5 (9.4)	0.34
Vital signs				
SBP (mmHg)	155.0 (138.8–170.0)	155.0 (138.5–170.0)	157.0 (139.0–170.5)	0.62
DBP (mmHg)	88.0 (78.0–96.0)	87.0 (76.0–97.0)	89.0 (81.5–95.0)	0.19
Heart rate (/min)	78.0 (70.0–87.0)	77.0 (68.0–86.0)	84.0 (75.0–94.0)	<0.01
Neurological status				
Hunt‐Hess grade 3–5	50 (16.1)	30 (11.7)	20 (37.7)	<0.01
WFNS grade 3–5	33 (10.6)	18 (7.0)	15 (28.3)	<0.01
Laboratory tests				
WBC (×109/L)	11.4 (9.4–14.3)	11.2 (9.3–13.9)	12.9 (10.1–15.8)	0.03
Lymphocyte (×109/L)	1.0 (0.7–1.3)	1.0 (0.7–1.3)	0.8 (0.7–1.1)	<0.01
Neutrophil (×109/L)	10.0 (7.7–12.7)	9.9 (7.6–12.4)	11.6 (8.9–14.2)	<0.01
Monocyte (×109/L)	0.4 (0.3–0.5)	0.4 (0.3–0.5)	0.5 (0.2–0.7)	0.09
RBC (×109/L)	4.5 (4.1–4.8)	4.5 (4.1–4.8)	4.5 (4.1–4.9)	0.90
Hb (g/L)	137.0 (127.0–148.0)	136.0 (127.0–148.0)	139.0 (125.5–149.0)	0.98
PLT (×109/L)	228.0 (195.0–272.0)	227.0 (195.0–269.0)	234.0 (195.0–282.0)	0.40
CRP (mg/L)	3.4 (1.2–5.6)	2.9 (1.1–5.6)	5.6 (2.5–10.8)	<0.01
FDP (mg/L)	2.7 (1.6–5.0)	2.4 (1.5–4.3)	3.3 (2.5–10.6)	<0.01
D‐dimer (mg/L)	1.0 (0.6–1.9)	0.9 (0.0–1.6)	1.5 (0.8–4.3)	<0.01
PT (s)	11.3 (10.8–11.8)	11.3 (10.8–11.8)	11.5 (10.8–12.1)	0.40
APTT (s)	27.4 (25.9–29.1)	27.4 (25.9–29.1)	27.2 (25.8–29.2)	0.74
Fbg (g/L)	3.0 (2.6–3.5)	3.0 (2.6–3.4)	3.1 (2.6–3.6)	0.11
TT (s)	14.6 (13.9–15.3)	14.6 (13.9–15.3)	14.7 (13.6–15.3)	0.41
Potassium (mmol/L)	3.8 (3.5–4.0)	3.8 (3.5–4.0)	3.7 (3.6–3.9)	0.14
Sodium (mmol/L)	137.4 (135.6–139.2)	137.4 (135.7–139.7)	137.5 (135.1–139.6)	0.90
Chlorine (mmol/L)	103.7 (101.5–105.8)	103.7 (101.7–105.8)	103.6 (100.5–105.5)	0.39
Glucose (mmol/L)	7.6 (6.5–9.1)	7.4 (6.4–8.8)	8.7 (6.8–10.5)	<0.01
BUN (mmol/L)	4.6 (3.8–5.4)	4.5 (3.7–5.3)	5.0 (4.3–6.7)	<0.01
Cr (μmol/L)	54.6 (47.4–64.8)	54.4 (47.2–64.0)	55.0 (48.4–70.2)	0.30
eGFR	112.5 (104.6–122.4)	113.2 (105.9–123.5)	107.6 (94.5–114.9)	<0.01
ALT (U/L)	17.4 (13.0–26.0)	17.1 (12.5–25.3)	19.0 (14.6–30.5)	0.03
AST (U/L)	19.0 (15.5–24.1)	18.7 (15.1–23.7)	21.4 (16.8–28.0)	<0.01
ALB (g/L)	42.8 (40.4–44.6)	42.9 (40.7–44.6)	42.2 (40.1–43.9)	0.23
TBIL (μmol/L)	11.2 (8.7–15.8)	11.2 (8.5–15.4)	11.9 (9.6–17.2)	0.15
DBIL (μmol/L)	5.2 (4.1–6.8)	5.1 (4.1–6.3)	5.8 (4.7–8.3)	0.02
IBIL (μmol/L)	6.1 (4.5–9.1)	6.1 (4.5–9.0)	6.1 (5.0–9.2)	0.50
CK‐MB (ng/mL)	1.7 (1.1–2.5)	1.6 (1.0–2.4)	2.0 (1.4–2.8)	0.02
cTNI (ng/mL)	0.004 (0.001—0.010)	0.003 (0.001–0.008)	0.008 (0.003–0.084)	<0.01
Aneurysm location				0.79
Anterior cerebral artery	95 (30.6)	76 (29.6)	19 (35.8)	
Internal carotid artery	130 (41.9)	110 (42.8)	20 (37.7)	
Middle cerebral artery	53 (17.1)	45 (17.5)	8 (15.1)	
Posterior circulation	32 (10.3)	26 (10.1)	6 (11.3)	
Aneurysm morphology				0.05
Single‐sac with smooth margin	73 (23.5)	66 (25.7)	7 (13.2)	
Single‐sac with irregular margin	76 (24.5)	57 (22.2)	19 (35.8)	
Aneurysm with a daughter sac	77 (24.8)	67 (26.1)	10 (18.9)	
Multilobulated aneurysm	84 (27.1)	67 (26.1)	17 (32.1)	
Multiple aneurysm	71 (22.9)	59 (23.0)	12 (22.6)	0.96
Modified Fisher grade 3–4	112 (36.1)	76 (29.7)	36 (67.9)	<0.01
Treatment				<0.01
Coiling	142 (45.8)	133 (51.8)	9 (17.0)	
Clipping	133 (42.9)	108 (42.0)	25 (47.2)	
Conservative treatment	35 (11.3)	16 (6.2)	19 (35.8)	

*Note*: Continuous variables are expressed as means ± (SD) or medians (IQR).

Abbreviations: ALB, albumin; ALT, alanine aminotransferase; APTT, activated partial thromboplastin time; AST, aspartate aminotransferase; BUN, blood urea nitrogen; CK‐MB, creatine kinase isoenzyme; Cr, creatinine; CRP, C‐reaction protein; cTNI, cardiac troponin I; DBIL, direct bilirubin; DBP, diastolic blood pressure; eGFR, estimated glomerular filtration rate; Fbg, fibrinogen; FDP, fibrin degradation products; Hb, hemoglobin; IBIL, indirect bilirubin; PLT, platelet; PT, prothrombin time; RBC, red blood cell; SBP, systolic blood pressure; TBIL, total bilirubin; TT, thrombin time; WBC, white blood cell; WFNS, World Federation of Neurosurgical Societies.

### 3.2 Feature selection and model development

**FIGURE 1:**
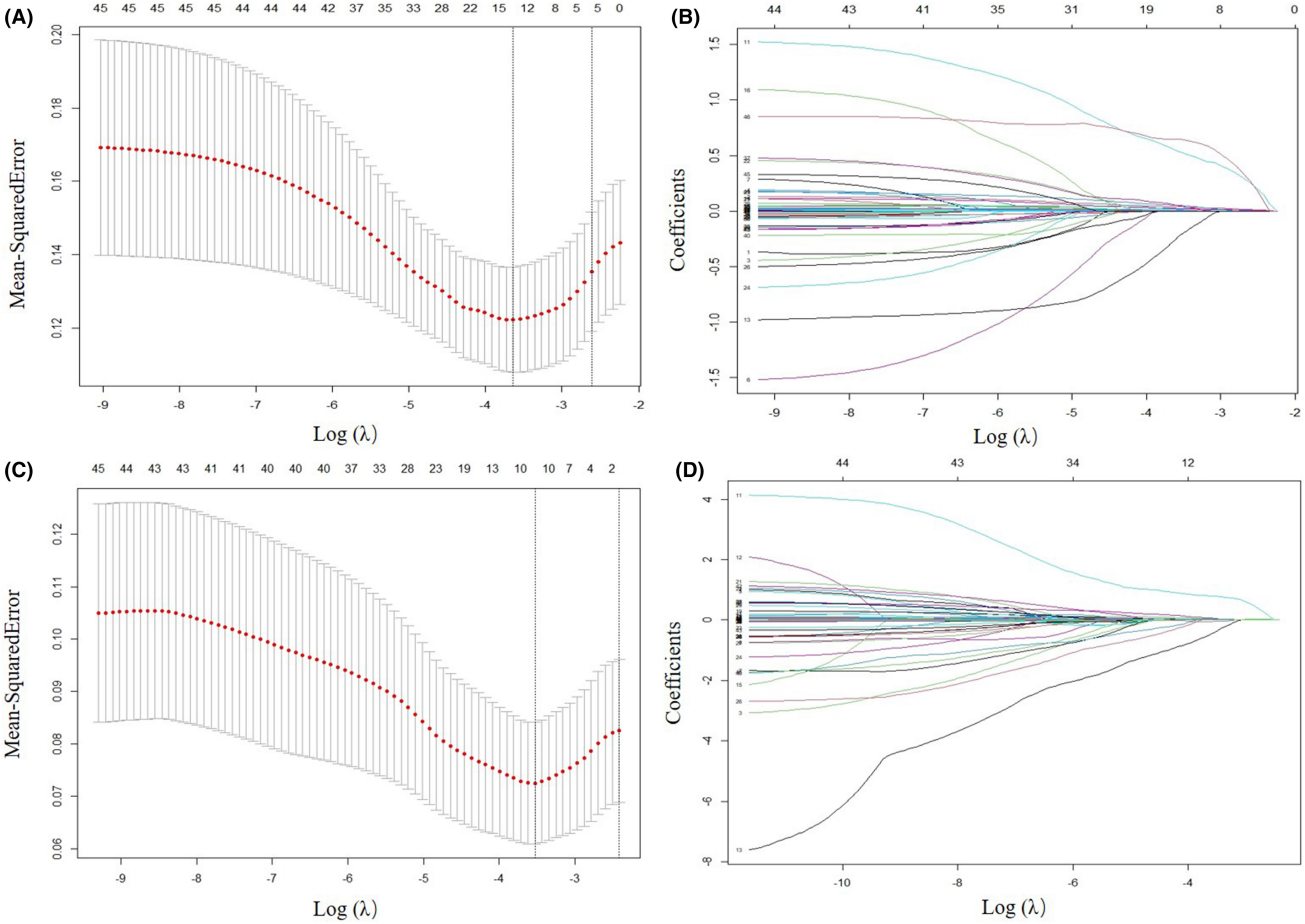
Selection of potential factors to predict 3‐month poor functional outcome (A, B) or all‐cause death (C, D) by LASSO regression analysis. (A, C) Five‐fold cross‐validation and minimum error criteria were applied to identify the optimal penalization coefficient. The left vertical line represents the optimal λ values when applying the minimum error criteria, while the right vertical line represents the optimal λ values when applying the one standard error of the minimum criteria. (B, D) LASSO coefficient profile plots against the log (λ). Optimal λ based on the minimum error criteria was used to select variables with nonzero coefficients.

Least absolute shrinkage and selection operator regression analysis was applied to select the potential predictors. In terms of 3‐month poor functional outcome, an optimal λ of 0.026 was adopted based on the minimum error criterion (Figure 1A). Therefore, a total of 46 variables were reduced to 15 variables according to the optimal λ (Figure 1B). These predictors with nonzero coefficients were age, diastolic blood pressure, heart rate, Hunt‐Hess grade, lymphocyte, neutrophil, platelet, CRP, D‐dimer, glucose, BUN, ALT, ALB, DBIL, and WFNS grade. Similarly, regarding 3‐month death, ten predictors with non‐zero coefficients were screened based on an optimal λ of 0.029 (Figure 1C,D). These predictors were age, heart rate, Hunt‐Hess grade, neutrophil, lymphocyte, CRP, D‐dimer, BUN, AST, and treatment methods.

Subsequently, multivariable logistics regression analysis incorporating factors with nonzero coefficients was performed to identify the independent variables associated with poor functional outcome or all‐cause death. The result demonstrated that age [adjusted OR (95% CI) = 1.070 (1.035–1.105), *p* < 0.001], heart rate [adjusted OR (95% CI) = 1.034 (1.009–1.059), *p* = 0.007], Hunt‐Hess grade [adjusted OR (95% CI) = 4.321 (1.796–10.392), *p* = 0.001], lymphocyte [adjusted OR (95% CI) = 0.294 (0.108–0.799), *p* = 0.016], platelet [adjusted OR (95% CI) = 1.007 (1.001–1.013), *p* = 0.024], CRP [adjusted OR (95% CI) = 1.069 (1.011–1.130), *p* = 0.019], and DBIL [adjusted OR (95% CI) = 1.186 (1.017–1.382), *p* = 0.029] were all significantly associated with 3‐month poor functional outcome (Table 2). While the independent predictors for 3‐month death were age [adjusted OR (95% CI) = 1.101 (1.042–1.163), *p* = 0.001], lymphocyte [adjusted OR (95% CI) = 0.093 (0.018–0.497), *p* = 0.005], neutrophil [adjusted OR (95% CI) = 1.322 (1.124–1.556), *p* = 0.001], CRP [adjusted OR (95% CI) = 1.085 (1.012–1.164), *p* = 0.021], AST [adjusted OR (95% CI) = 1.071 (1.029–1.116), *p* = 0.001], and endovascular coiling [adjusted OR (95% CI) = 0.047 (0.007–0.328), *p* = 0.002] (Table 3).

**TABLE 2:** Multivariable logistic regression analysis for 3‐month poor functional outcome.

Variables	β	Adjusted OR	95% CI	*p*
Age	0.068	1.070	1.035–1.105	<0.001
Heart rate	0.033	1.034	1.009–1.059	0.007
Hunt‐Hess grade 3–5	1.463	4.321	1.796–10.392	0.001
Lymphocyte	−1.223	0.294	0.108–0.799	0.016
Platelet	0.007	1.007	1.001–1.013	0.024
CRP	0.067	1.069	1.011–1.130	0.019
DBIL	0.170	1.186	1.017–1.382	0.029

Abbreviations: CI, confidence interval; CRP, C‐reactive protein; DBIL, direct bilirubin; OR, odd ratio.

**TABLE 3:** Multivariable logistic regression analysis for 3‐month all‐cause death.

Variables	β	Adjusted OR	95% CI	*p*
Age	0.096	1.101	1.042–1.163	0.001
Lymphocyte	−2.371	0.093	0.018–0.497	0.005
Neutrophil	0.279	1.322	1.124–1.556	0.001
CRP	0.082	1.085	1.012–1.164	0.021
AST	0.069	1.071	1.029–1.116	0.001
Treatment				<0.001
Clipping		1 (reference)		
Coiling	−3.054	0.047	0.007–0.328	0.002
Conservative treatment	0.673	1.960	0.527–7.293	0.316

Abbreviations: CRP, C‐reaction protein; AST, aspartate aminotransferase; OR, odd ratio; CI, confidence interval.

**FIGURE 2:**
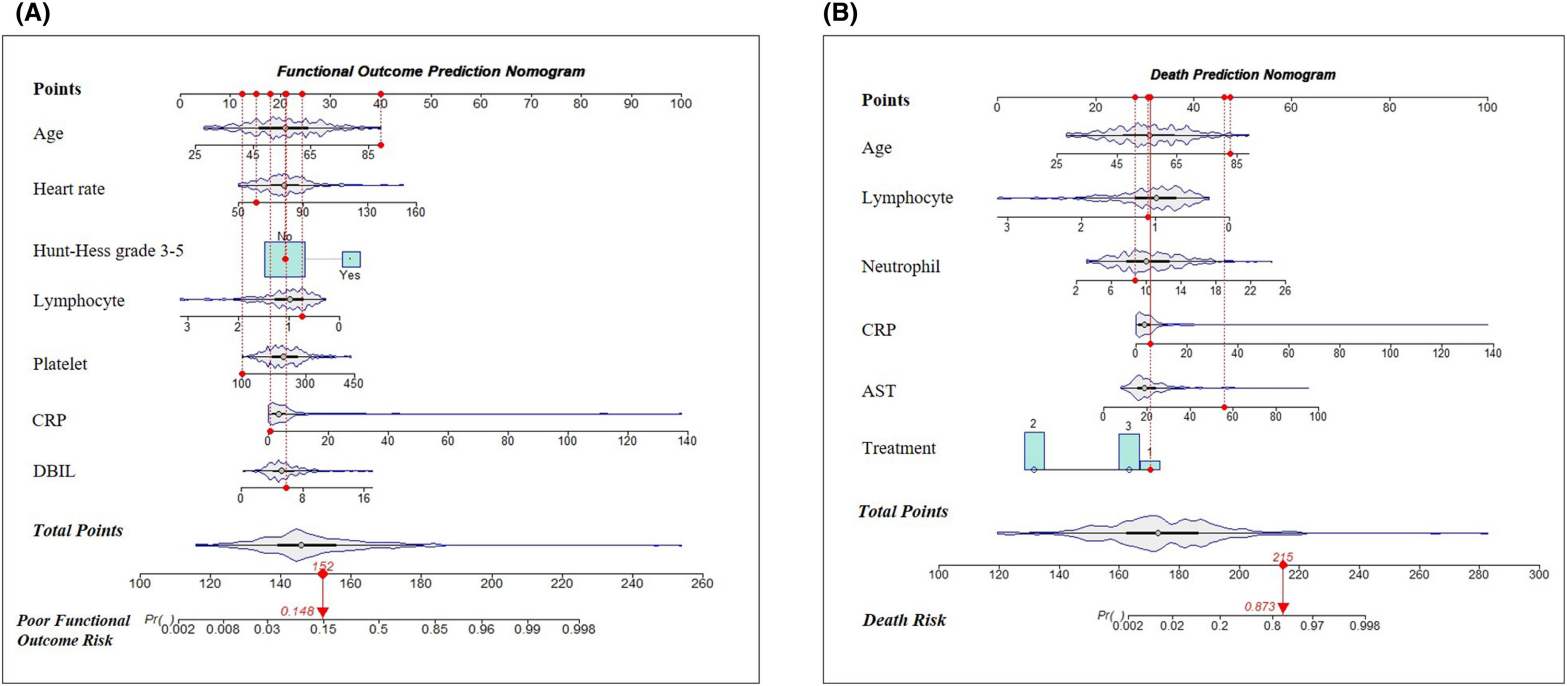
Nomogram models for predicting 3‐month poor functional outcome (A) or all‐cause death (B). AST, aspartate aminotransferase; CRP, C‐reactive protein; DBIL, direct bilirubin.

Based on the result of multivariable logistics regression analysis, two nomogram models were constructed to better visualize the estimation of the individual risk for poor functional outcome or all‐cause death (Figure 2A,B). Each predictor was projected upward to an exact point and the total sum of the points in the nomogram model were converted into an individual risk of the 3‐month poor functional outcome or all‐cause death.

### 3.3 Model performance and validation

**FIGURE 3:**
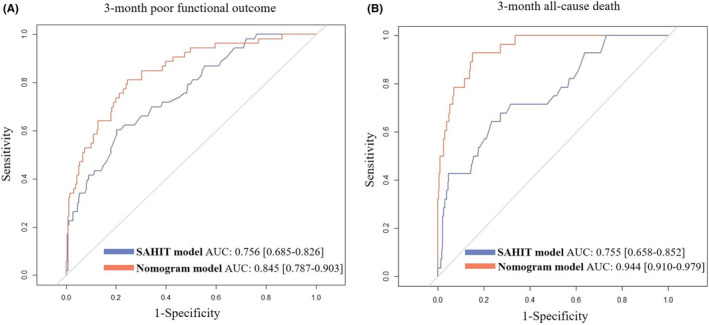
ROC curves of the two newly developed nomogram models and SAHIT models for predicting 3‐month poor functional outcome (A) or all‐cause death (B). ROC: receiver operating characteristic; AUC: area under the curve; SAHIT: Subarachnoid Hemorrhage International Trialists'.

The ROC curve analysis showed that the nomogram model to predict 3‐month poor functional outcome yielded greater AUC values (0.845, 95% CI: 0.787–0.903) than that of the core SAHIT model (AUC: 0.756, 95% CI: 0.685–0.826, *p* = 0.002) (Figure 3A). In terms of 3‐month all‐cause death, similar results were also discovered for the superior discrimination power of nomogram model (AUC: 0.944, 95% CI: 0.910–0.979) than did the SAHIT model (AUC: 0.755, 95% CI: 0.658–0.852, *p* < 0.001) (Figure 3). Compared with the core SAHIT models, the calibration curves of both two nomogram models displayed a more satisfactory agreement between prediction and actual observation for 3‐month poor functional outcome or all‐cause death (Figure S1). Furthermore, the decision curve analysis was displayed to explore the clinical usefulness of the nomogram models. The result showed that both two nomogram models revealed superior overall net benefits compared with the SAHIT model (Figure S2).

The two nomogram models were internally validated by 1000 bootstraps validation method. The bias‐corrected C index for predicting 3‐month poor functional outcome and all‐cause death was 0.827 and 0.927, respectively, which all exhibited excellent discrimination capacities.

External validation of two nomogram models was also conducted. A total of 208 eligible aSAH patients were included in the external validation cohort. The majority of the patients were female (63%) with a mean age of 56.8 years old. Poor functional outcome after 3 months was observed in 19.7% of the patients, while the mortality rate was 10.6%. No significant difference was identified between the derivation cohort and external validation cohort (Table S2). The result of ROC analysis, calibration curves, and decision curve analysis based on the external validation cohort were demonstrated in Figure S3. The nomogram model predicting poor functional outcome showed an AUC of 0.795 (95% CI: 0.716–0.873). A similar result was identified in the nomogram model to predict all‐cause death, with an AUC of 0.811 (95% CI: 0.707–0.915). The calibration plots of the two nomogram models also presented an acceptable agreement between the outcome prediction and actual observation. In terms of decision curve analysis, nomogram models to predict poor functional outcome or all‐cause death all provided good clinical utility.

## 4 DISCUSSION

In the present study, we developed and validated two novel nomogram models for predicting poor functional outcome or all‐cause death at 3 months in patients with aSAH. Both two models demonstrated favorable performance and predictive accuracy in derivation and externally validation cohorts.

### 4.1 The Nomogram model to predict poor functional outcomes

A total of seven pronounced risk factors, including age, Hunt‐Hess grade, heart rate at presentation, lymphocyte, CRP, platelet, and DBIL levels, were finally incorporated into the nomogram model to predict 3‐month poor functional outcome. Age was reported to be a major significant predictor for poor outcome in many literatures.^18^, ^19^, ^20^ This could be explained by the fact that the elderly is more likely to have poor vascular conditions,^21^ less ability to recover from the initial bleeding and prone to develop delayed cerebral ischemia, and other complications.^8^, ^21^ Apart from the advanced age, neurological grade at admission such as Hunt‐Hess grade, is usually applied in clinical settings to evaluate the severity and prognosis of aSAH patients.^22^ The Hunt‐Hess grade categorizes the neurological status into five levels according to patients' symptoms and level of consciousness.^23^ Although this grading system has showed superior performance for predicting poor functional outcome, it is not reliable enough due to its subjective nature of each parameter in this system, especially applying in intubated patients.^24^ Moreover, Hunt‐Hess grade performs poorly at demarcating between adjacent grades due to the ambiguous defined scales of consciousness,^25^ which makes it difficult to apply for further stratified management and targeted treatment. Hence, it's crucial to consider other relatively objective parameters that can better reflect the pathogenesis of aSAH for further predictions and treatment. Recently, the role of early brain injury and its associated peripheral‐blood‐based biomarkers to predict prognosis after aSAH is worthy of recognition.^26^, ^27^ Higher levels of CRP could be a good reflection of acute neuroinflammation response after aSAH occurs, subsequently resulting in early brain injury and unfavorable functional outcomes.^28^, ^29^ The mechanisms of this process might include blood–brain barrier disruption, neuronal death, and white matter injury.^30^, ^31^ Also, bleeding in the subarachnoid space initiates platelet activation and aggregation,^32^ which further triggers early brain injury owing to microthrombosis, cerebral tissue ischemia, and death.^33^, ^34^ Therefore, elevated platelet counts associated with poor functional outcome adds to the evidence that thrombosis formation might participate in the mechanism of early brain injury.^35^ In addition, bilirubin is a hemoglobin degradation product after aSAH and also takes part in early brain injury.^36^ Blood clots in the subarachnoid space induce free radical formation, which can subsequently act on bilirubin and produce bilirubin oxidation products.^36^ These toxic products ultimately damage vascular smooth muscle cells and affect the blood flow of the brain tissue,^37^ aggravating the brain injury. On the contrary, lymphocytes play an important role in anti‐inflammatory response and immune surveillance.^38^, ^39^ Therefore, patients with lower level of lymphocytes might imply that they are in an immunosuppressive state, making it tough for them to recover from the early brain injury.^40^

### 4.2 The Nomogram model to predict all‐cause death

Variables associated with all‐cause death at 3 months after aSAH onset are partially overlapped with those associated with disability, such as lymphocyte and CRP, which explains the shared pathogenesis of death and disability after aSAH. Additional parameters important for death prediction include neutrophil, AST levels and treatment methods. Aneurysmal bleeding promotes neuroinflammatory responses, which is characterized by elevated neutrophil counts.^30^, ^38^ Increased levels of neutrophils take crucial part in early brain injury and poor prognosis through a variety of mechanisms, including activation of reactive oxygen species, disruption of endothelial cell conjunctions, and increased blood brain barrier permeability.^41^, ^42^ In this study, we also found that elevated AST levels were one of the major risk factors for 3‐month death in patients with aSAH. The underlying mechanisms of this association remain unclear and still need future studies to uncover. We speculate that increased AST levels may indicate concurrent subclinical liver disease,^43^ which may be closely related to vascular inflammation, coagulation dysfunction, and impaired endothelial function.^44^

When it comes to treatment methods, our study has demonstrated that endovascular coiling was associated with patient survival. As it known to all, surgical treatment can significantly improve the prognosis of patients with aSAH compared with conservative treatment. However, how to choose between endovascular coiling and surgical clipping remains controversial. The International Subarachnoid Aneurysm Trial (ISAT) compared the safety and effectiveness of endovascular coiling and surgical clipping in 2143 patients with ruptured aneurysms.^45^ Results have shown that endovascular coiling was more likely to be associated with independent survival at 1 year and 10 years compared with surgical clipping.^46^ However, the risk of late rebleeding was higher after endovascular coiling than after surgical clipping.^47^ Concerning the inclusion criteria of ISAT trials that requires patients suitable for both surgical treatments, patients with post‐circulation aneurysms or larger wide‐necked aneurysms may have been excluded from the ISAT cohorts. Therefore, other study still tried to explore the benefits of different surgical methods. A meta‐analysis by Li et al. showed that there was no significant difference in terms of 1‐year mortality between the two surgical methods.^48^ Different results between literatures may be primarily explained by different characteristics of study populations (e.g., the severity of disease, the location of aneurysms). In ISAT trials, about 88% of patients were low‐grade aSAH patients (WFNS grade 1–2), and 95% of aneurysms located in the anterior circulation.^45^ Similarly, in our study, low‐grade aSAH patients accounted for 89%, and nearly 90% of the patients had aneurysms located in the anterior circulation. However, in the meta‐analysis mentioned above,^48^ the percentage of high‐grade aSAH patients and aneurysms located in the posterior circulation were higher than our study and ISAT trials, so the advantage of endovascular coiling for improving survival may be diminished. All in all, endovascular coiling may be a good choice not only for patients who are suitable for both the two surgical methods but also for patients with low preoperative grading scales and aneurysms located in the anterior circulation.

### 4.3 Comparisons between the SAHIT model

Recently, Jaja et al. developed the SAHIT prediction models for poor functional outcome or death after 3 months in patients with aSAH.^4^ The models were constructed based on a large population gathered from several clinical trials and have been validated rigorously.^49^ Thus, the SAHIT models are the most widely accepted model to predict outcomes after aSAH. However, the predictors just explained 23%–31% of the variability in outcomes,^4^ implying other factors may take part in the outcome prediction and they were not fully captured from the SAHIT models. Furthermore, the predictor hypertension in the SAHIT models were not well‐defined^3^ and WFNS grade incorporated in the SAHIT models performed poorly in patients with adjacent grade scales.^25^ On the contrary, our nomogram models take other objective blood‐based biomarkers that could reflect the pathogenesis of aSAH into account, which yielded a better performance compared with the core SAHIT models. Another weakness of the SAHIT models is that it developed on trial data. Trials may select specified study population, which can differ from the general population in real practice.^3^, ^9^ And randomization to the studied intervention could alter the expectations of clinicians and patients, which may affect the outcomes eventually.^9^ In contrast, our current models were based on patient data derived from real‐world practice in a modern‐day cohort, showing an advantage compared with the core SAHIT models. In addition, the SAHIT models can only be utilized through an online calculator, making it difficult to be widely used to some extent. In the current study, nomogram plots were applied to present our models, which is easy to use in various clinical settings.

### 4.4 Strengths and limitations

There are several strengths in the current study. First, the identified parameters not only can be readily measured and obtained from routine examinations but also convenient to use in acute aSAH phase without the need for much experience to evaluate imaging scales and consider the subsequent complications during treatment course. Second, the LASSO regression applied in our study has advantages to lower data dimensionality and reduce multicollinearity between variables, thus, it is performed well in selecting the potential significant parameters. Moreover, nomogram excels in simplicity over traditional analysis utilizing odd ratios and can help to better visualize the model to generate an individual risk of poor outcomes, making it beneficial for physicians to apply in clinical practice.

However, there are also some limitations in our study to be recognized. First, our study was conducted in a single tertiary center with a relatively small sample size, which might introduce selection bias. A multicenter and large study is required to validate our findings in the future. Second, we only evaluated outcomes at 3 months after symptom onset in patients with aSAH, future studies should focus on the long‐term prognosis of aSAH patients. Moreover, external validation from a different cohort is still needed to further verify the generalizability of our models.

### 4.5 Clinical implications

The nomogram prediction models in the present study could assist in providing patient‐centered information of poor prognosis and help clinicians to communicate effectively with patients and their relatives. More importantly, using the readily obtained variables on admission, our prediction models help meet the demand for identifying and screening patients at high risk of poor outcomes quickly at an early stage of aSAH. Therefore, clinicians can pay more attention to these high‐risk patients and promptly conduct personalized therapy to prevent further deterioration and significantly improve prognosis of these patients. Furthermore, another potential clinical significance of our study is that the nomogram models contain predictors that can reflect the process of early brain injury. Hence, it provides meaningful insight into future studies to explore treatments that target neuroinflammatory response, microthrombosis formation, toxic effects of degradation products and so on.

## 5 CONCLUSION

In the present study, nomogram models constructed for predicting 3‐month poor functional outcome or death after aSAH demonstrated good discrimination, accuracy, and clinical usefulness. The predictor items in these two nomogram models are not only easy to obtain upon admission but also can reflect the pathogenesis of aSAH to a certain extent. Therefore, these two models can help physicians to identify patients at risk, guide decision‐making at an early stage, and provide new directions for future studies to explore the novel treatment targets.

## AUTHOR CONTRIBUTIONS

SL collected the original data, performed the statistics analysis, interpreted the data, and drafted the original manuscript. JZ, NL, and DW contributed to data interpretation. XZ designed the research and handled funding and supervision. All authors read and approved the final manuscript.

## FUNDING INFORMATION

This study was supported by the Chinese Academy of Medical Sciences Innovation Fund for Medical Sciences (2019‐I2M‐5‐029), Beijing Municipal Committee of Science and Technology (Z201100005620010), Beijing Hospitals Authority Innovation Studio of Young Staff Funding Support (code: 202112), and the Ministry of Finance of the People's Republic of China (issued by Finance and Social Security [2015] Document No. 82; [2016] Document No. 50; [2017] Document No. 72; [2018] Document No. 48; [2019] Document No. 77; [2020] Document No. 75; [2021] Document No. 84, [Ministry of Finance]).

## CONFLICT OF INTEREST STATEMENT

None.

## Supporting information


Figure S1.

Figure S2.

Figure S3.

Table S1.

Table S2.

## Data Availability

The data that support the findings of this study are available from the corresponding author upon reasonable request.
