# Supplementary figures and images for: Predictive nomogram models for unfavorable prognosis after aneurysmal subarachnoid hemorrhage: Analysis from a prospective, observational cohort in China
Source: CNS Neurosci Ther. 2023 Jun 8;29(11):3567–78. doi: 10.1111/cns.14288 (PMC10580355; doi:10.1111/cns.14288)

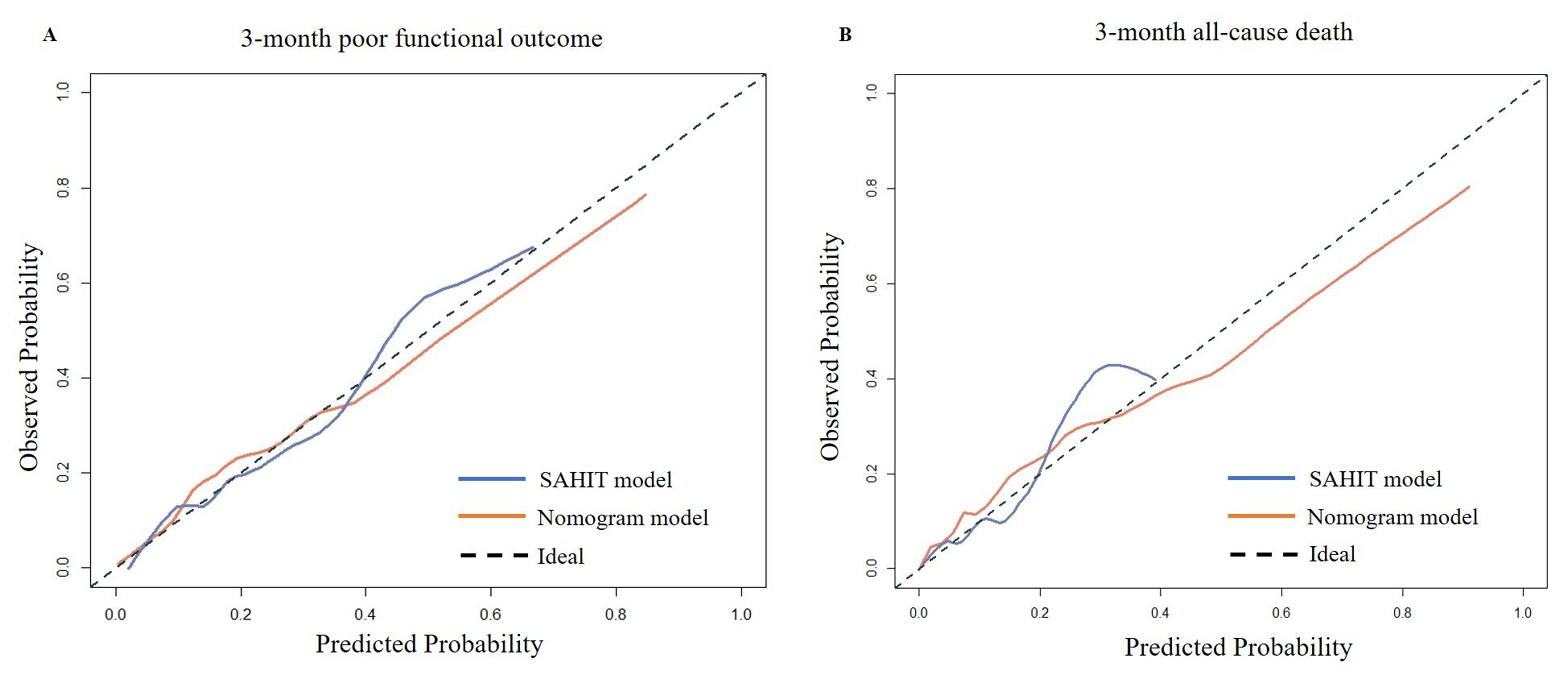

Supplement: Supplementary file 1 — Figure S1. [file CNS-29-3567-s004.zip › Figure S1_Suppinfo_1.tif]

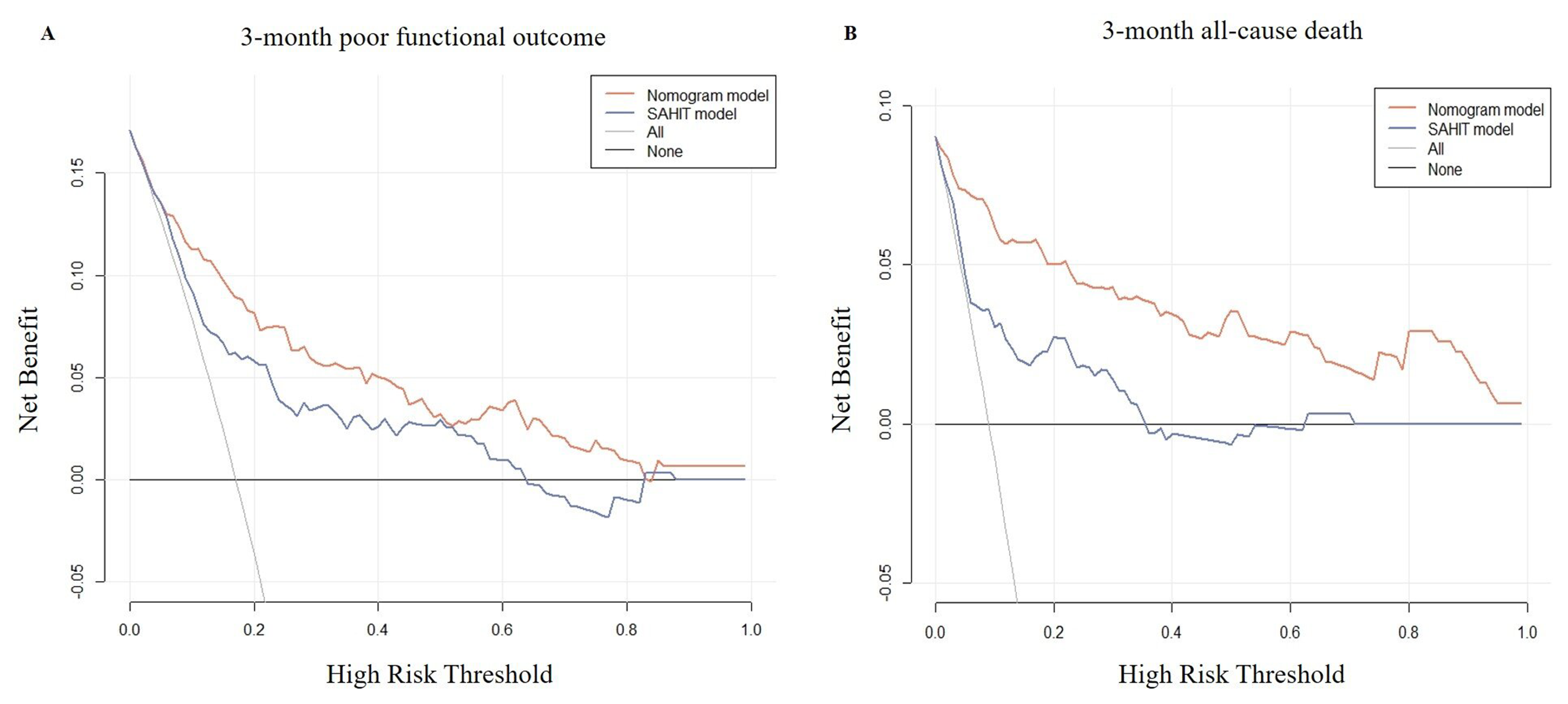

Supplement: Supplementary file 2 — Figure S2. [file CNS-29-3567-s001.zip › Figure S2_Suppinfo_1.tif]

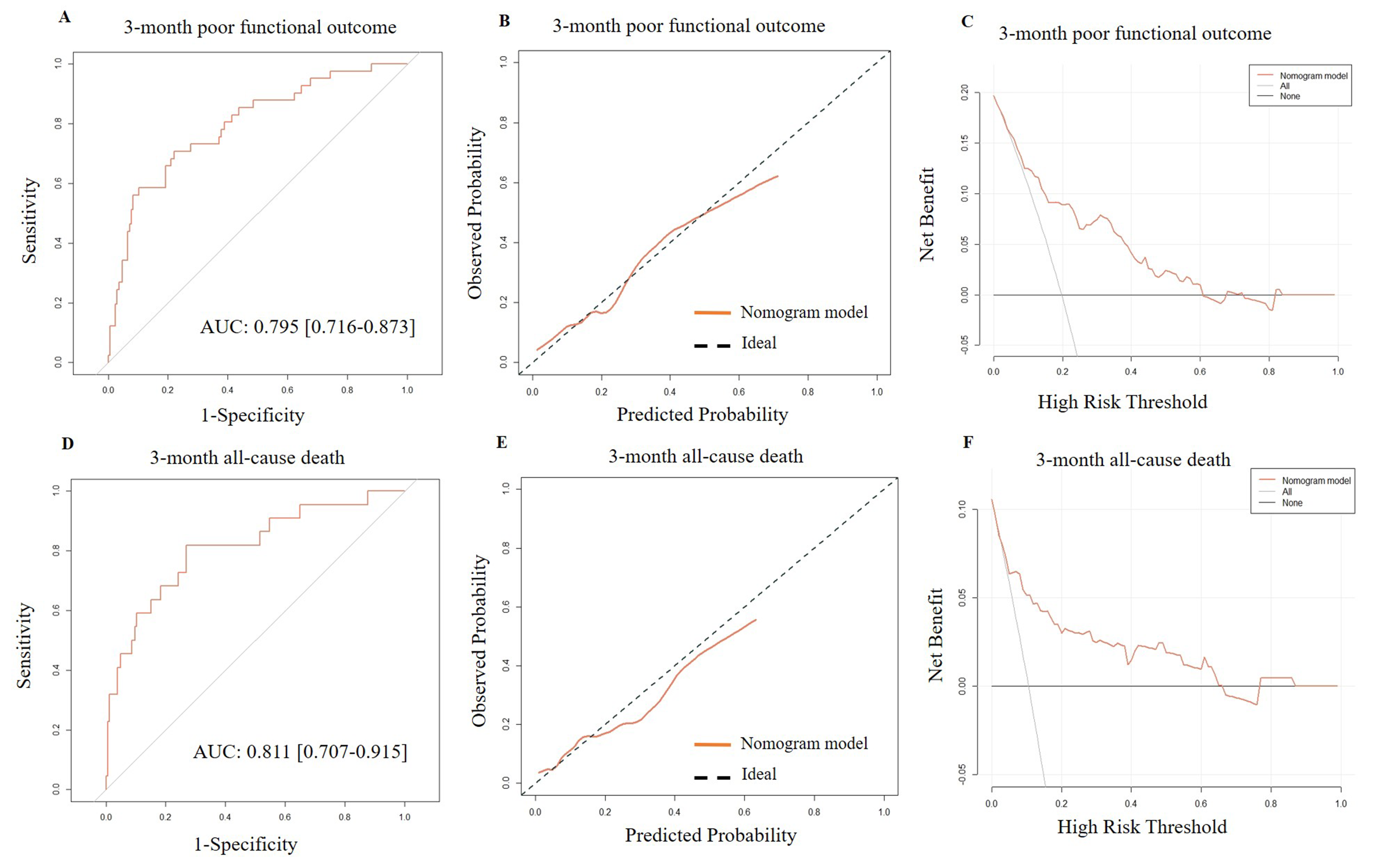

Supplement: Supplementary file 3 — Figure S3. [file CNS-29-3567-s005.zip › Figure S3_Suppinfo_1.tif]
