# Supplementary material for: Predictive nomogram models for unfavorable prognosis after aneurysmal subarachnoid hemorrhage: Analysis from a prospective, observational cohort in China
Source: CNS Neurosci Ther. 2023 Jun 8;29(11):3567–78. doi: 10.1111/cns.14288 (PMC10580355; doi:10.1111/cns.14288)
Supplement: Supplementary file 4 — Table S1. [file CNS-29-3567-s002.docx]

Table 1. Baseline characteristics of the study population grouped by 3-month death

|  | Total (n=310) | Alive (n=282) | Death (n=28) | P |
| --- | --- | --- | --- | --- |
| Age (years) | 56.1±12.4 | 55.2±12.0 | 65.6±13.1 | ＜0.01 |
| Female sex | 200（64.5） | 181（64.2） | 19（67.9） | 0.70 |
| **History** |  |  |  |  |
| Hypertension | 191（61.6） | 173（61.3） | 18（64.3） | 0.76 |
| Diabetes mellitus | 31（10.0） | 28（9.9） | 3（10.7） | 0.75 |
| Coronary heart disease | 25（8.1） | 24（8.5） | 1（3.6） | 0.71 |
| Current smoking | 53（17.1） | 50（17.7） | 3（10.7） | 0.44 |
| Alcohol | 42（13.5） | 40（14.2） | 2（7.1） | 0.40 |
| **Vital signs** |  |  |  |  |
| SBP (mmHg) | 155.0 (138.8-170.0) | 155.0 (139.0-170.0) | 155.0 (135.0-170.0) | 0.73 |
| DBP (mmHg) | 88.0（78.0-96.0） | 88.0（77.0-96.3） | 88.0（80.3-95.0） | 0.53 |
| Heart rate (/min) | 78.0（70.0-87.0） | 78.0（69.0-86.0） | 83.5（75.3-101.5） | ＜0.01 |
| **Neurological status** |  |  |  |  |
| Hunt-Hess grade 3-5 | 50（16.1） | 36（12.8） | 14（50.0） | ＜0.01 |
| WFNS grade 3-5 | 33（10.6） | 25（8.9） | 8（28.6） | ＜0.01 |
| **Laboratory tests** |  |  |  |  |
| WBC (×109/L) | 11.4（9.4-14.3） | 11.2（9.3-13.9） | 13.8（10.4-16.6） | ＜0.01 |
| Lymphocyte (×109/L) | 1.0（0.7-1.3） | 1.0 (0.7-1.3) | 0.7 (0.5-1.0) | ＜0.01 |
| Neutrophil (×109/L) | 10.0（7.7-12.7） | 9.9 (7.6-12.4) | 12.5 (9.2-15.6) | ＜0.01 |
| Monocyte (×109/L) | 0.4（0.3-0.5） | 0.4 (0.3-0.5) | 0.5 (0.3-0.7) | 0.01 |
| RBC (×109/L) | 4.5（4.1-4.8） | 4.5（4.1-4.8） | 4.5（4.0-4.8） | 0.82 |
| Hb (g/L) | 137.0（127.0-148.0） | 136.0（127.0-148.0） | 139.0（125.3-150.3） | 0.97 |
| PLT(×109/L) | 228.0（195.0-272.0） | 227.0（195.0-269.5） | 233.5（186.5-282.5） | 0.54 |
| CRP (mg/L) | 3.4（1.2-5.6） | 3.1（1.1-5.6） | 5.6（3.4-13.6） | ＜0.01 |
| FDP (mg/L) | 2.7（1.6-5.0） | 2.6（1.6-4.5） | 3.3（2.5-17.7） | ＜0.01 |
| D-dimer (mg/L) | 1.0（0.6-1.9） | 1.0（0.6-1.8） | 1.6（0.9-10.5） | ＜0.01 |
| PT (s) | 11.3（10.8-11.8） | 11.3（10.8-11.8） | 11.5（10.7-12.1） | 0.92 |
| APTT (s) | 27.4 (25.9-29.1) | 27.4 (25.9-29.1) | 27.4 (25.8-29.6) | 0.82 |
| Fbg (g/L) | 3.0（2.6-3.5） | 3.0（2.6-3.4） | 3.3（2.9-3.6） | 0.03 |
| TT (s) | 14.6（13.9-15.3） | 14.6（13.9-15.3） | 14.6（13.6-15.3） | 0.47 |
| Potassium (mmol/L) | 3.8（3.5-4.0） | 3.8（3.5-4.0） | 3.7（3.6-3.8） | 0.11 |
| Sodium (mmol/L) | 137.4（135.6-139.2） | 137.4（135.6-139.6） | 137.9（135.0-140.8） | 0.46 |
| Chlorine (mmol/L) | 103.7（101.5-105.8） | 103.6（101.4-105.7） | 104.3（101.9-106.8） | 0.56 |
| Glucose (mmol/L) | 7.6（6.5-9.1） | 7.5（6.4-8.9） | 8.7（7.3-9.8） | ＜0.01 |
| BUN (mmol/L) | 4.6（3.8-5.4） | 4.5（3.7-5.3） | 5.2（4.5-7.0） | ＜0.01 |
| Cr (umol/L) | 54.6（47.4-64.8） | 54.6（47.4-64.7） | 55.0（47.5-70.2） | 0.58 |
| eGFR | 112.5（104.6-122.4） | 112.6 (105.4-122.6) | 106.7 (89.8-117.3) | 0.02 |
| ALT (U/L) | 17.4（13.0-26.0） | 17.3（12.7-25.9） | 19.0（14.7-33.1） | 0.07 |
| AST (U/L) | 19.0（15.5-24.1） | 18.8（15.1-23.2） | 27.0（18.4-36.7） | ＜0.01 |
| ALB (g/L) | 42.8（40.4-44.6） | 42.9（40.7-44.6） | 41.9（39.8-44.5） | 0.26 |
| TBIL (umol/L) | 11.2（8.7-15.8） | 11.3（8.6-15.6） | 10.6（8.8-16.6） | 0.78 |
| DBIL (umol/L) | 5.2（4.1-6.8） | 5.2（4.1-6.5） | 5.3（3.8-8.4） | 0.36 |
| IBIL (umol/L) | 6.1（4.5-9.1） | 6.2（4.5-9.1） | 5.7（4.7-9.1） | 0.86 |
| CK-MB (ng/ml) | 1.7（1.1-2.5） | 1.6（1.1-2.4） | 2.5（1.4-5.4） | ＜0.01 |
| cTNI (ng/ml) | 0.004（0.001—0.010） | 0.004（0.001-0.009） | 0.007（0.004-0.166） | ＜0.01 |
| **Aneurysm location** |  |  |  | 0.50 |
| Anterior cerebral artery | 95（30.6） | 86（30.5） | 9（32.1） |  |
| Internal carotid artery | 130（41.9） | 119（42.2） | 11（39.3） |  |
| Middle cerebral artery | 53（17.1） | 50（17.7） | 3（10.7） |  |
| Posterior circulation | 32（10.3） | 27（9.6） | 5（17.9） |  |
| **Aneurysm morphology** |  |  |  | 0.56 |
| Single-sac with smooth margin | 73（23.5） | 69（24.5） | 4（14.3） |  |
| Single-sac with irregular margin | 76（24.5） | 67（23.8） | 9（32.1） |  |
| Aneurysm with a daughter sac | 77（24.8） | 69（24.5） | 8（28.6） |  |
| Multilobulated aneurysm | 84（27.1） | 77（27.3） | 7（25.0） |  |
| Multiple aneurysm | 71（22.9） | 66（23.4） | 5（17.9） | 0.51 |
| Modified Fisher grade 3-4 | 112（36.1） | 92（32.6） | 20（71.4） | ＜0.01 |
| **Treatment** |  |  |  | ＜0.01 |
| Coiling | 142（45.8） | 138（48.9） | 4（14.3） |  |
| Clipping | 133（42.9） | 123（43.6） | 10（35.7） |  |
| Conservative treatment | 35（11.3） | 21（7.4） | 14（50.0） |  |

Continuous variables are expressed as means ± (SD) or medians (IQR).

SBP: systolic blood pressure; DBP: diastolic blood pressure; WFNS: World Federation of Neurosurgical Societies; WBC: white blood cell; RBC: red blood cell; Hb: hemoglobin; PLT: platelet; CRP: C-reaction protein; FDP: fibrin degradation products; PT: prothrombin time; APTT: activated partial thromboplastin time; Fbg: fibrinogen; TT: thrombin time; BUN: blood urea nitrogen; Cr: creatinine; eGFR: estimated glomerular filtration rate; ALT: alanine aminotransferase; AST: aspartate aminotransferase; ALB: albumin; TBIL: total bilirubin; DBIL: direct bilirubin; IBIL: indirect bilirubin; CK-MB: creatine kinase isoenzyme; cTNI: cardiac troponin I
