# Supplementary material for: Predictive nomogram models for unfavorable prognosis after aneurysmal subarachnoid hemorrhage: Analysis from a prospective, observational cohort in China
Source: CNS Neurosci Ther. 2023 Jun 8;29(11):3567–78. doi: 10.1111/cns.14288 (PMC10580355; doi:10.1111/cns.14288)
Supplement: Supplementary file 5 — Table S2. [file CNS-29-3567-s003.docx]

Table S2. Comparisons of baseline characteristics in derivation and external validation cohort.

|  | Derivation cohort  (n=310) | External validation cohort  (n=208) | P |
| --- | --- | --- | --- |
| Age (years) | 56.1±12.4 | 56.8±11.8 | 0.51 |
| Female sex | 200（64.5） | 131 (63.0) | 0.72 |
| **History** |  |  |  |
| Hypertension | 191（61.6） | 111 (53.4) | 0.06 |
| Diabetes mellitus | 31（10.0） | 23 (11.1) | 0.70 |
| Coronary heart disease | 25（8.1） | 14 (6.7) | 0.57 |
| Current smoking | 53（17.1） | 24 (11.5) | 0.08 |
| Alcohol | 42（13.5） | 13 (6.3) | ＜0.01 |
| **Vital signs** |  |  |  |
| SBP (mmHg) | 155.0 (138.8-170.0) | 153.0 (140.0-165.8) | 0.62 |
| DBP (mmHg) | 88.0（78.0-96.0） | 85.5 (77.0-94.0) | 0.36 |
| Heart rate (/min) | 78.0（70.0-87.0） | 78.5 (70.0-88.0) | 0.48 |
| **Neurological status** |  |  |  |
| Hunt-Hess grade 3-5 | 50（16.1） | 32 (15.4) | 0.82 |
| WFNS grade 3-5 | 33（10.6） | 13 (6.3) | 0.09 |
| **Laboratory tests** |  |  |  |
| WBC (×109/L) | 11.4（9.4-14.3） | 12.1 (9.8-15.0) | 0.14 |
| Lymphocyte (×109/L) | 1.0（0.7-1.3） | 1.1 (0.8-1.5) | 0.03 |
| Neutrophil (×109/L) | 10.0（7.7-12.7） | 10.4 (7.9-13.3) | 0.29 |
| Monocyte (×109/L) | 0.4（0.3-0.5） | 0.5 (0.3-0.6) | ＜0.01 |
| RBC (×109/L) | 4.5（4.1-4.8） | 4.6 (4.3-4.8) | 0.02 |
| Hb (g/L) | 137.0（127.0-148.0） | 141.0 (131.3-151.0) | ＜0.01 |
| PLT(×109/L) | 228.0（195.0-272.0） | 237.0 (204.0-272.0) | 0.25 |
| CRP (mg/L) | 3.4（1.2-5.6） | 2.6 (1.1-5.8) | 0.33 |
| FDP (mg/L) | 2.7（1.6-5.0） | 2.2 (1.5-4.5) | 0.05 |
| D-dimer (mg/L) | 1.0（0.6-1.9） | 0.9 (0.6-1.7) | 0.73 |
| PT (s) | 11.3（10.8-11.8） | 11.3 (10.8-11.8) | 0.88 |
| APTT (s) | 27.4 (25.9-29.1) | 28.0 (26.5-29.8) | ＜0.01 |
| Fbg (g/L) | 3.0（2.6-3.5） | 3.1 (2.8-3.5) | 0.13 |
| TT (s) | 14.6（13.9-15.3） | 14.4 (13.7-14.9) | ＜0.01 |
| Potassium (mmol/L) | 3.8（3.5-4.0） | 3.8 (3.5-4.1) | 0.31 |
| Sodium (mmol/L) | 137.4（135.6-139.2） | 137.8 (135.8-139.8) | 0.19 |
| Chlorine (mmol/L) | 103.7（101.5-105.8） | 104.2 (102.0-106.6) | 0.03 |
| Glucose (mmol/L) | 7.6（6.5-9.1） | 7.3 (6.4-9.1) | 0.45 |
| BUN (mmol/L) | 4.6（3.8-5.4） | 4.5 (3.7-5.8) | 0.95 |
| Cr (umol/L) | 54.6（47.4-64.8） | 55.6 (47.8-66.5) | 0.23 |
| eGFR | 112.5（104.6-122.4） | 113.2 (102.8-119.7) | 0.37 |
| ALT (U/L) | 17.4（13.0-26.0） | 17.0 (12.7-24.6) | 0.56 |
| AST (U/L) | 19.0（15.5-24.1） | 20.0 (16.0-25.0) | 0.15 |
| ALB (g/L) | 42.8（40.4-44.6） | 42.2 (40.0-44.4) | 0.16 |
| TBIL (umol/L) | 11.2（8.7-15.8） | 11.3 (8.6-) | 0.90 |
| DBIL (umol/L) | 5.2（4.1-6.8） | 5.3 (4.1-6.8) | 0.83 |
| IBIL (umol/L) | 6.1（4.5-9.1） | 6.0 (4.4-9.1) | 0.74 |
| CK-MB (ng/ml) | 1.7（1.1-2.5） | 1.4 (0.9-2.3) | 0.08 |
| cTNI (ng/ml) | 0.004（0.001—0.010） | 0.004 (0.001-0.011) | 0.99 |
| **Aneurysm location** |  |  | 0.18 |
| Anterior cerebral artery | 95（30.6） | 51 (24.5) |  |
| Internal carotid artery | 130（41.9） | 82 (39.4) |  |
| Middle cerebral artery | 53（17.1） | 45(21.6) |  |
| Posterior circulation | 32（10.3） | 30 (14.4) |  |
| **Aneurysm morphology** |  |  | 0.18 |
| Single-sac with smooth margin | 73（23.5） | 49 (23.6) |  |
| Single-sac with irregular margin | 76（24.5） | 68 (32.7) |  |
| Aneurysm with a daughter sac | 77（24.8） | 46 (22.1) |  |
| Multilobulated aneurysm | 84（27.1） | 45 (21.6) |  |
| Multiple aneurysm | 71（22.9） | 49 (23.6) | 0.86 |
| Modified Fisher grade 3-4 | 112（36.1） | 111 (53.4) | ＜0.01 |
| **Treatment** |  |  | 0.47 |
| Coiling | 142（45.8） | 84 (40.4) |  |
| Clipping | 133（42.9） | 97 (46.6) |  |
| Conservative treatment | 35（11.3） | 27 (13.0) |  |

Continuous variables are expressed as means ± (SD) or medians (IQR).

SBP: systolic blood pressure; DBP: diastolic blood pressure; WFNS: World Federation of Neurosurgical Societies; WBC: white blood cell; RBC: red blood cell; Hb: hemoglobin; PLT: platelet; CRP: C-reaction protein; FDP: fibrin degradation products; PT: prothrombin time; APTT: activated partial thromboplastin time; Fbg: fibrinogen; TT: thrombin time; BUN: blood urea nitrogen; Cr: creatinine; eGFR: estimated glomerular filtration rate; ALT: alanine aminotransferase; AST: aspartate aminotransferase; ALB: albumin; TBIL: total bilirubin; DBIL: direct bilirubin; IBIL: indirect bilirubin; CK-MB: creatine kinase isoenzyme; cTNI: cardiac troponin I
